# Measuring the resilience of criminogenic ecosystems to global disruption: A case-study of COVID-19 in China

**DOI:** 10.1371/journal.pone.0240077

**Published:** 2020-10-14

**Authors:** Hervé Borrion, Justin Kurland, Nick Tilley, Peng Chen

**Affiliations:** 1 Department of Security and Crime Science, University College London, London, United Kingdom; 2 School of Criminal Justice, Forensic Science, and Security, Institute for Advanced Analytics in Security, University of Southern Mississippi, Hattiesburg, MS, United States of America; 3 School of Policing and Information Engineering, People’s Public Security University of China, Beijing, China; US Army Engineer Research and Development Center, UNITED STATES

## Abstract

This paper uses resilience as a lens through which to analyse disasters and other major threats to patterns of criminal behaviour. A set of indicators and mathematical models are introduced that aim to quantitatively describe changes in crime levels in comparison to what could otherwise be expected, and what might be expected by way of adaptation and subsequent resumption of those patterns. The validity of the proposed resilience assessment tool is demonstrated using commercial theft data from the COVID-19 pandemic period. A 64 per cent reduction in crime was found in the studied city (China) during an 83-day period, before daily crime levels bounced back to higher than expected values. The proposed resilience indicators are recommended as benchmarking instruments for evaluating and comparing the global impact of COVID-19 policies on crime and public safety.

## 1. Introduction

Crime patterns vary by place and can fluctuate over short time periods. Studies on the temporal patterns of crime have focused on seasonal patterns such as time of year, day of week, holidays, and hours of darkness [1–3]. Event generated perturbations in crime patterns have been less studied. These can be brought about by large-scale disruption to the public safety of communities and to the infrastructure upon which they rely. While exceptional in scale, such events are not rare. Across the globe, natural disasters (epidemics, hurricanes, volcanic eruptions, earthquakes, cyclones, etc.), man-made catastrophes (terrorist attacks, industrial accidents, fires, etc.), and even mega sporting events (Summer and Winter Olympics, Football World Cup, etc.) that radically disrupt everyday life are not uncommon [4]. COVID-19 comprises a major source of global disruption, whose scale is unprecedented.

Reports both in the media and by police already suggest that COVID-19 is having a substantial and immediate impact on criminal behaviour across diverse jurisdictions [5, 6]. With the important exception of domestic violence, most types of offence appear to have fallen steeply. The longer-term consequences for crime patterns are clearly not yet known at the time of writing but we anticipate crime rates for offences that dropped will likely increase once lockdown measures are lifted. This paper provides a methodological framework and a set of metrics for analysing the effect of COVID-19 and associated (individual and formal) responses on crime, within and across cities. The framework used is grounded in ‘resilience’ theory and the metrics draw upon what might be expected on the basis of analogous disruptive events of different kinds, and their immediate and longer-term impacts on institutions and behaviours as they normalise.

We begin by explaining our rationale for using resilience as a lens through which to analyse the immediate disruption of criminal activity and what might be expected in the longer term assuming the criminal behaviour has resilient qualities. Next, we describe indicators and mathematical models devised to measure the characteristics of the disruption to, and resilience of the criminogenic ecosystem. Finally, we briefly illustrate the application of these metrics using empirical data from an anonymous city in China (M1-city) that unlike much of the developed world, has gone through a complete wave of the COVID-19 pandemic and the majority of routine activities have now resumed.

## 2. Background

### 2.1 Resilience

Resilience in the social-ecological context relates to the ability of human agents to learn and innovate, to manage shocks, and to also find new trajectories for communities and social-technical systems. More specifically, it focuses on (a) the amount of change a system can undergo (and, therefore, the amount of disturbance it can sustain) while still retaining the same controls on structure and function); (b) the degree to which the system is capable of self-organization and (c) the degree to which the system can build the capacity to learn, adapt, and ultimately offer a ‘soft landing’ for the organisation or system [7, 8]. Systems are resilient to the extent to which they can adapt to and survive an event that threatens to disrupt them. Such threats can be internal or external. They can arise as a result of human actions (such as war) or natural disasters (such as hurricanes and disease outbreak). The threats can apply, for example, to commercial organisations, families, economies, religious groups, or political systems. Resilience studies explore ways in which (eco)systems are initially thrown off course, then adjust to disruptive events and ultimately revert to their previous state, fail gracefully, or some new normal [9–11].

Much national planning has been concerned with resilience in the face of expected threats including those that spring from pandemics of the kind being experienced in the case of COVID-19 [12]. Most discussions of what is and can be done relate to the public interest in maintaining organisations and behaviours in the face of potentially disruptive threats with the majority of this work focusing on natural hazards and human error. Similarly there has been a focus on security and the ways in which threats can be absorbed if encountered or anticipated in advance and therefore geared up for when they occur [13–15]. The latter is sometimes referred to as ‘antifragility’, following Taleb [16]. Strong organisations are those that have increased capacity to withstand successive threats by building on lessons learned from each and preparing accordingly [17]. Studying the impact of the 7/7 terrorist attack, Cox et al. [18], for example, attempted to measure resilience as a function of the speed and extent to which the London Underground was able to adapt and resume its core functions. They considered the number of daily journeys as a measure of performance and found that resilience was a function of not just the public transport system, but also individual users.

### 2.2 Disruption, resilience and patterns of criminal behaviour

In contrast to the literature on disruptive threats to public interest and how they might be mitigated, here we are concerned specifically with changes in criminal behaviour patterns generated as a consequence of the disruption created by exceptional events.

There is a small literature on what has been observed in the short term in relation to patterns of criminal behaviour following disruptive natural and man-made disasters. In what follows previously established disaster categories and peril terminology for operational purposes are utilised to classify studies conducted in the criminological and sociological literature [19]. Summarised in Tables 1 and 2 previous studies found no one-to-one relationship between major types of disaster and their crime patterns consequences. However, they do show that effects are produced. A more nuanced discussion is required to fully unpack what happens within individual disasters and responses to them to understand the short and longer term impacts on crime and public safety.

**Table 1:** Main categories of natural disasters and findings about crime pattern consequences.

Group	Definition	Type	Crime Went Up	Crime Went Down
**Geophysical**	Events originating from solid earth	Earthquake, Volcano, Mass Movement (dry)	[20]–Murder	[25]–All crime
			[21]–DV^1^	[21]–All crime
			[22]–DV	[22]–All crime
			[23]–DV	[23]–All crime
			[24]–Child Abuse	[26]–Property crime
**Meteorological**	Events caused by short‐lived/small to meso-scale atmospheric processes (in the spectrum from minutes to days)	Storm (hurricane, typhoons)	[24]–Child Abuse	[28]–All crime
			[27]–Residential and commercial burglary	[29]–All crime
**Hydrological**	Events caused by deviations in the normal water cycle and/or overflow of bodies of water caused by wind set‐up	Flood, Mass Movement (wet)	[30]–Contractor fraud	[31]–All crime
				[32]–All crime
**Climatological**	Events caused by long‐lived/meso- to macro-scale processes (in the spectrum from intra‐seasonal to multi‐decadal climate variability)	Extreme Temperature, Drought, Wildfire	[33]–DV and dowry murder	[33]–All crime
**Biological**	Disaster caused by the exposure of living organisms to germs and toxic substances	Epidemic, Insect Infestation, Animal Stampede	[34]–Violent and property crime; stranger homicides	
**Extra-terrestrial**	Events caused by extra-terrestrial bodies or phenomena	Meteorites, Asteroids, Solar Flares		

^1^ DV is the abbreviation for domestic violence.

**Table 2:** Main categories of man-made disasters and findings about crime pattern consequences.

Group	Definition	Type	Crime Went Up	Crime Went Down
**Malicious**	Events maliciously caused by individuals or groups (incl. offenders, non-states actors and states).	Terrorist attacks, sabotage, arson, riots, threats posed by the presence or actions of armed groups	[35]–Looting and property crime	
			[36]–Looting and property crime	
**Accidental**	Unforeseen events not maliciously caused by individuals or groups (accidents) and technological failures.	Technological failure, human error	[37]–All crime	
**Legitimate**	Events intentionally organised non-malicious individuals or groups for a different purpose.	Sporting event, strike, political rally	[38]–Violent offences	[40]–All crime
				[38]–All crime
			[39]–Property and violent offences	

Taking COVID-19 as an example, the following discussion sketches a theoretical model, rooted in notions of resilience, of what would be expected in relation to crime pattern changes following a pandemic.

There is good reason to believe that many patterns of criminal behaviour have been disrupted by the COVID-19 pandemic and/or subsequent public-health measures. Indeed, between 1 January 2020 and 5 May 2020, police data shows there has been a significant and unusual drop in rape, theft, and assault to name a few in many cities including New York (-29%; -10%; -5%), Chicago (-15%; -7%; -22%) and Los Angeles (-25%; -25%; -4%) when compared to counts from the previous year [6, 41, 42]. These changes are not altogether surprising as crime is a function of patterned opportunity that arises from the routine activity of everyday human life [43]. Moreover, the fundamental organising principles of society revolve around tasks that require people to act jointly in collective efforts related, for example, to work, school, and various forms of leisure. As restrictions inhibiting the movement potential of individuals to engage in the activities that underpin community structure are altered, so too will be the rhythm associated with them. Indeed, even precautionary behaviours in anticipation of a pending disruptive event can disturb this periodicity. In turn, the ecological conditions required for many different crime categories shaped by the coordination of these rhythms will be thrown into disarray.

Take residential burglary, for example. This requires a motivated offender to identify a suitable target in the absence of a guardian that can intervene. The scarcity of guardian-free residences during the lockdowns introduced in response to COVID-19 coupled with offenders’ inability to ‘forage’, as doing so may draw unnecessary attention thus undermining the anonymity desired, combine to increase the risk of committing a burglary. However, this pattern will likely be temporary and levels of residential burglary and other crimes will return to previous (or higher) levels for several reasons. For instance, some offenders may adapt to these new conditions, finding new and innovative ways to take advantage of drastically reduced footfall and the natural surveillance it provides along with other elements of the criminogenic ecosystem. Alternatively, a return to ecological equilibrium, brought about by lifting stringencies on behaviour, may lead back to the previous rhythms that naturally afforded offenders opportunities they could exploit.

## 3. Measuring and modelling resilience

Fine-grained data that can be used to capture empirical nuances of resilience across crime types and sub-types during and following COVID-19 will soon become available. At that point, a common set of indicators will be necessary to perform a global analysis of crime during the pandemic period.

### 3.1 Measuring resilience

Numerous analytic methods and indicators have been used in different sectors to evaluate and measure resilience [44]. Shin et al. [45] reviewed quantitative approaches for measuring the resilience of water infrastructure systems and evaluated 21 indicators for assessing major features of resilience. However, continuous variables (quantity and flow of water) cannot be used in the analysis of crime. Furthermore, concepts found in supply and demand problems (such as reserve capacity and availability) are not directly applicable to public safety problems because offenders and victims have competing goals.

A useful review from the transportation sector by Sun et al. [46] pointed to metrics that are more relatable to criminogenic ecosystems by making a distinction between functionality and resilience metrics:

Functionality metrics (or performance measures) provide information about the (static or dynamic) state of a system, whereas resilience metrics are used to evaluate the continued functionality of a system during a disturbance. The former include metrics related to the topological features of a system as well as metrics related to traffic flow and system capacity such as travel time, throughput, and congestion. In the public safety domain, *crime incidence* (number of crimes) or the *amount of harm* (e.g., financial loss, environmental damage) caused by these events can serve as functionality metrics [47].

**Fig 1:**
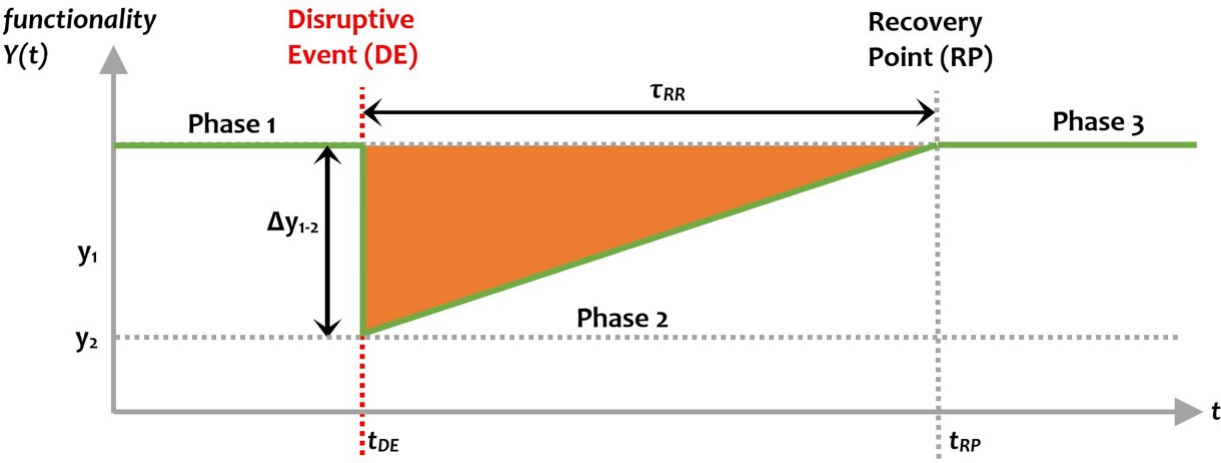
Resilience triangle adapted from Bruneau et al [48].

Along the same public safety vein, resilience metrics can use the above-mentioned metric values to quantify a system’s functional resilience. In other disciplines a common approach has been to compare the performance measure *before* and *after* a disruptive event, or to quantify the social and economic impact. The resilience triangle is a tool proposed by Bruneau et al. (2003) for the purpose of modelling the loss of resilience after the occurrence of disruption to a system (Fig 1). The results are normalised, enabling the loss in performance, Δ*y*(*t*) = *y*_*1—*_*y*(*t*), to be more readily interpreted in relation to loss in the worst case scenario, max(Δ*y*(*t*)) = 1.

More specifically, the resilience triangle provides a measure of both the loss of functionality in a system in the wake of disaster (*t*_*DE*_) and the amount of time required for a system to recover. Several metrics have been proposed that include the recovery time, the slope of the recovery speed (α), the length of the recovery path, which in combination quantify the area of the ‘resilience triangle’ (Eq 1) along with the ‘resilience index’ that captures the average functionality level until the end of the study period (*t*_*h*_) (Eq 2):

$$R_{L}=\int_{t_{DE}}^{t_{RP}}[1-Y(t)]dt,$$ (1)

$$R(t_{h})=\frac{\int_{t_{DE}}^{t_{h}}Y(t)dt}{t_{h}-t_{DE}},$$ (2)

where, *Y*(*t*) is the system functionality at time *t*, *t*_*DE*_ is the time when the disruptive event occurs, *t*_*RP*_ is the recovery point, and *t*_*h*_ is the time horizon of the analysis.

Other metrics have been created to follow the evolution of resilience over time. For example, Ouyang and Wang [49] proposed a metric that compares actual functionality (*Y*_*A*_) with a target functionality (*Y*_*T*_*)* between *t*_*0*_ (the start of the prevention stage) and *t*_*h*_. (Eq 3):

$$R(t_{h})=\frac{\int_{t_{0}}^{t_{h}}Y_{A}(t)dt}{\int_{t_{0}}^{t_{h}}Y_{T}(t)dt}.$$ (3)

Socioeconomic resilience metrics have also been used to capture the economic impact of disruption. Focusing on short term impact, Rose [50] presented a metric called Direct Static Economic Resilience (DSER) that represents ‘the extent to which the estimated direct output reduction deviates from the likely maximum potential reduction given an external shock’ (Eq 4):

$$DSER=\frac{\%\Delta Y-\%\Delta Y^{m}}{\%\Delta Y^{m}},$$ (4)

where, %Δ*Y*^*m*^ is the maximum percent change in direct output and %Δ*Y* is the (estimated) actual percent change in direct output.

### 3.2 Resilience indicators for crime analysis

The selection of an appropriate functionality metric or performance measure depends on the system under analysis. For a commercial organisation, the *Y(t)* function may be based on sales, profit, return on investment, and market share [51]. For criminals, different variables are required as metrics. A lone offender may be regarded as analogous to a sole tradesman; a small group of offenders to a small organisation; and a large criminal network (e.g., the mafia) to a multinational firm. In the case of common crimes such as burglary, most offenses are committed by single offenders or small groups of co-offenders [52]. Naturally, daily crime counts for a given area may be considered an appropriate performance metric in this case. An alternative, and closely linked to the economic models of resilience, would be the daily revenue generated from offenses [53].

**Fig 2:**
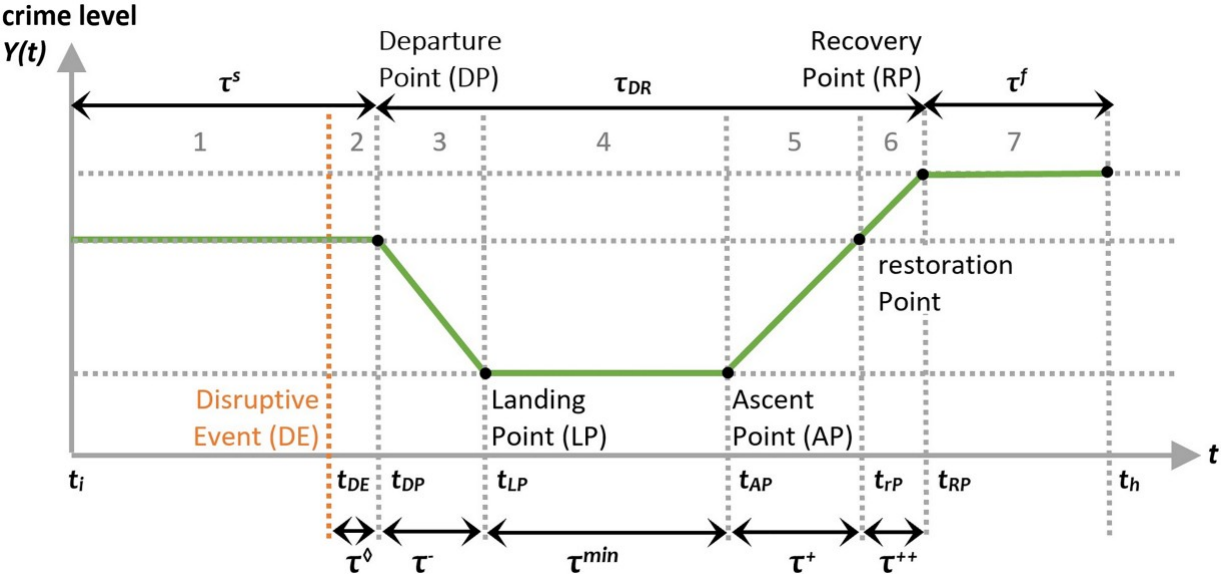
Crime levels following a disruptive event.

There are reasons to believe that crime-related performance measures during a pandemic period, or other disasters, may not match the idealised resilience triangle, but rather resemble the dipper-shaped function depicted in Fig 2.

This point is developed further in what follows:

1. Resilience curves often assume that functionality and performance start to decrease immediately after the disruptive event occurs. This may not always be the case with crime. As an example, the predicted increase in domestic abuse may only begin several days after households began to be confined to their homes. Consequently, it is reasonable to consider that there may be a lag (**τ**^◊^) between the disruptive event (*t*_*DE*_) and departure point (*t*_*DP*_) from the normal state of a system.
2. Resilience curves assume that the loss of functionality/performance happens *after* the disruptive event. While true for most systems, humans are often able to anticipate and pre-empt disrupting events [54]. COVID-19 prompted many people to begin stocking up on essential products. In turn, this anticipation by individual actors and the associated response to stock up on essential products appeared to generate, at least according to media reports, a series of physical and/or verbal assaults *prior* to a single observable case of COVID-19 [55]. For this reason, the analysis window also includes an Initial Phase, starting at *t*_*i*_, considered the earliest point when performance could have been affected by the disruptive event. The time between *t*_*i*_ and *t*_*DP*_ is referred to as **τ**^s.^
3. Resilience curves often represent loss of functionality as a quasi-instantaneous effect, such as when a light goes off during a blackout. This is, however, not always the case (c.f., [49, 50, 56]). While many systems might experience a sharp drop in functionality or performance, the criminogenic factors that influence crime frequency during exceptional times are unlikely to change instantaneously. In the case of pandemics, for example, it is known that public-health measures affect biological and socio-technical systems (i.e., virus, individuals, communities, infrastructures) gradually. Therefore, the damping period (**τ**^-^) starting with the departure point (*t*_*DP*_) and leading to the landing point (*t*_*LP*_) before the trough may last several days or even weeks.
4. Resilience curves commonly found in the literature generally assume that disruptive events are *punctual* instances (such as an earthquake) and that a recovery phase starts almost immediately thereafter. However, some disruptive events can transpire over a longer interval causing sustained disruption to the environment as noted in other recent resilience studies on this pandemic [57–59]. In the case of COVID-19, various different contagion prevention measures were introduced and enforced across several weeks in a staggered pattern as opposed to a singular instance (see [51]). For this reason, *t*_*i*_ should be regarded as the potential start of a disruptive *process*, with the maximum performance loss occurring over a sustained period (**τ**^min^) between what we refer to as the landing point (*t*_*LP*_) and ascent point (*t*_*AP*_).
5. The duration of the recovery phase (**τ**^R^) is also an interesting feature to observe as it provides information about the ability of criminogenic ecosystems to ‘bounce back’. Unlike the resilience triangle in Fig 1, it is possible that the system finds an alternative equilibrium point with a lower or higher crime level. In the case of the latter, a restoration Point (*t*_*rP*_) demarcates the time when performance returns to its normal level and further delineates two sub-phases: the pre-restoration phase (**τ**^+^) and the post-restoration phases (**τ**^++^). The duration of these phases provide additional information for understanding the trajectory of a new equilibrium.

Fluctuating rates are an additional motivation for adapting the resilience triangle to crime phenomena. More specifically, the assumption that underpins this particular indicator is the stationarity of performance under normal times, a condition violated by the inconstant patterning of crime events across time. To overcome this violation of assumption we propose indicators that take account of the difference between crime levels under two scenarios: with and without the disrupting event (Eq 5):

$$D(n)=Y_{F}(n)-Y_{A}(n),$$ (5)

where, *D*(*n*) is the difference between *Y*_*F*_(*n*) and *Y*_*A*_(*n*), the forecasted crime (under normal conditions) and actual crime time-series (daily crime counts), and *n* is the (temporal) index of the time-series.

The Relative Difference in crime levels, *RD*(*n*), represents the number of crimes that were not committed relative to the number of crimes expected to occur on any given day (Eq 6):

$$RD(n)=\frac{Y_{F}(n)-Y_{A}(n)}{Y_{F}(n)}.$$ (6)

*RD*(*n*) provides a useful measure to monitor the operating level (%) of offenders. However, it cannot be used directly to calculate cumulative losses in performance, that is the total number of offenses that did not occur. With varying forecasted crime levels (*Y*_*F*_), the difference, *D*(*n*), in crime levels is a more appropriate measure for this.

Another measure more closely aligned with the concept developed by Bruneau et al. [48] is the Normalised Difference in crime levels. *ND*(*n*) describes the difference in crime levels relative to the mean of the forecasted crime level (Eq 7):

$$ND(n)=\frac{Y_{F}(n)-Y_{A}(n)}{mean(Y_{F}(n))}.$$ (7)

**Fig 3:**
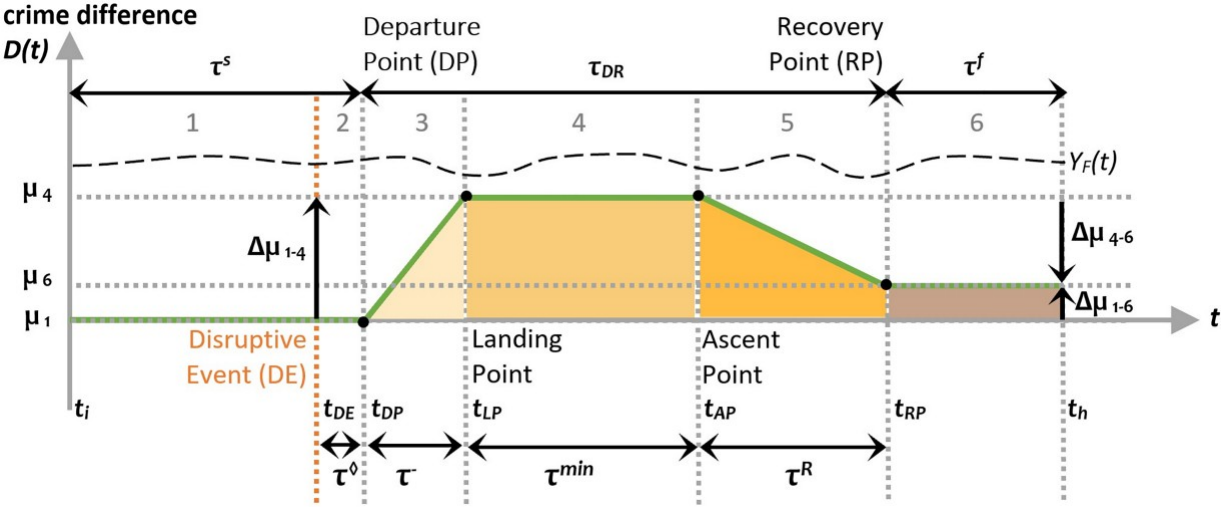
Loss in crime performance following a disruptive event (μ_1_<μ_6_).

**Fig 4:**
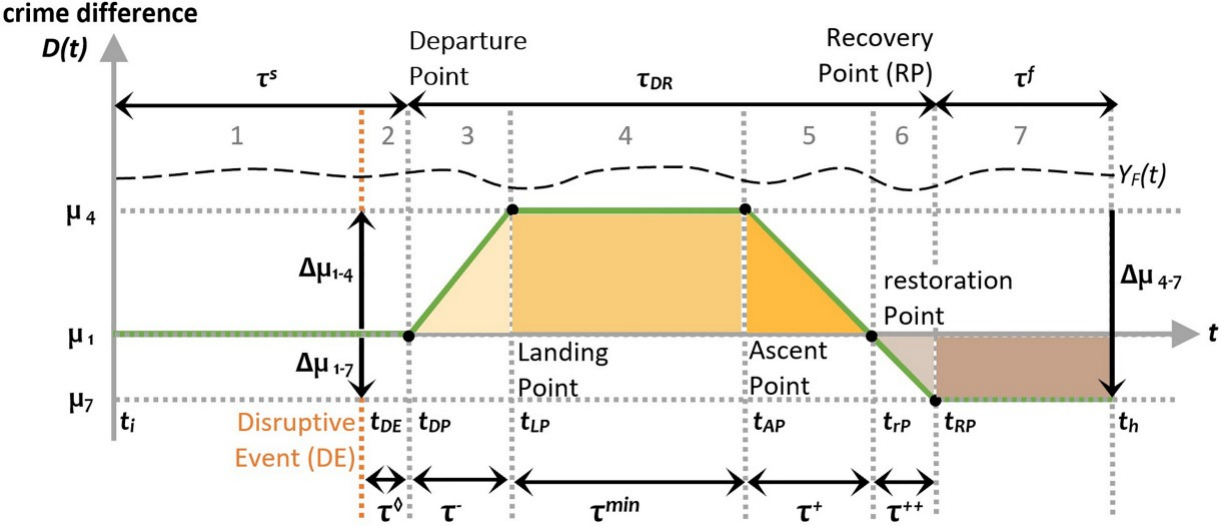
Loss in crime performance following a disruptive event (μ_1_≥μ_7_).

Represented in Figs 3 and 4, this non-performance measure (see [52]) can be used to quantitatively describe the resilience of different criminogenic ecosystems. In these two models, *t*_*i*,_ *t*_*DE*_, *t*_*DP*_, *t*_*LP*_, *t*_*AP*_, *t*_*rP*_, and *t*_*RP*_ correspond to the onset of the different phases described above.

The principal resilience indicator, the Normalised Difference Resilience Indicator (NDRI), is an estimate of crime reduction over the period of interest as a proportion of the total number of crimes expected in that period (Eq 8):

$$NDRI=\frac{\sum_{n_{i}}^{n_{h}}\left(Y_{F}(n)-Y_{A}(n)\right)}{\sum_{n_{i}}^{n_{h}}Y_{F}(n)},$$ (8)

where, *n*_*i*_ is the index of the initial point (the earliest reasonable opportunity for crime to change as a result of the disruptive event) and *n*_*h*_ is the index of the time horizon of the analysis.

Six associated indicators described in Eqs 9–14 can be used to characterise ecosystem resilience during the damping, trough, recovery and post-recovery phases, separately.

Similar to the NDRI, the Damping Phase Resilience Indicator (DPRI) focuses on the second phase of Figs 2 and 3. This indicator is concerned with the area of the lightest coloured orange triangle in the damping phase and can be calculated using Eq 9:

$$DPRI=\frac{\sum_{n_{DP}}^{n_{LP}-1}\left(Y_{F}(n)-Y_{A}(n)\right)}{\sum_{n_{DP}}^{n_{LP}-1}Y_{F}(n)}.$$ (9)

The Trough Phase Resilience Indicator (TPRI) is used to assess an ecosystem’s resilience. Represented in Eq 10, it quantifies the relative loss in performance sustained by the ecosystem during the third phase and relates to the rectangular orange coloured area in Fig 3:

$$TPRI=\frac{\sum_{n_{LP}}^{n_{AP}-1}\left(Y_{F}(n)-Y_{A}(n)\right)}{\sum_{n_{LP}}^{n_{AP}-1}Y_{F}(n)}.$$ (10)

The Recovery Phase Resilience Indicator (RPRI) in Eq 11 can be used to measure the ecosystem’s resilience during the recovery phase and corresponds to the brown coloured triangular area in Fig 3:

$$RPRI=\frac{\sum_{n_{AP}}^{n_{RP}-1}\left(Y_{F}(n)-Y_{A}(n)\right)}{\sum_{n_{AP}}^{n_{RP}-1}Y_{F}(n)}.$$ (11)

In the case where the crime level in this phase exceeds the pre-Disruptive Event (*t*_*DE*_) level (μ_7_≥μ_1_), two sub-indicators can be used to measure the ecosystem’s resilience during the recovery phase. They are, the pre- and post-restoration Phase Resilience Indicators (pre-rPRI and post-rPRI) in Eqs 12 and 13, and correspond to the coloured triangular areas in phases 5 and 6 in Fig 3:

$$pre-rPRI=\frac{\sum_{n_{AP}}^{n_{rP}-1}\left(Y_{F}(n)-Y_{A}(n)\right)}{\sum_{n_{AP}}^{n_{rP-1}}Y_{F}(n)},$$ (12)

$$post-rPRI=\frac{\sum_{n_{rP}}^{n_{RP}-1}\left(Y_{F}(n)-Y_{A}(n)\right)}{\sum_{n_{rP}}^{n_{RP}-1}Y_{F}(n)}.$$ (13)

The Posterior Phase Resilience Indicator (PPRI) estimates the relative loss in the new equilibrium phase when *t*_*h*_ ≥ *t*_*RP*_ (Eq 14). It corresponds to the brown coloured rectangular area in Fig 3:

$$PPRI=\frac{\sum_{n_{RP}}^{n_{h}}\left(Y_{F}(n)-Y_{A}(n)\right)}{\sum_{n_{RP}}^{n_{h}}Y_{F}(n)}.$$ (14)

## 4. COVID-19 case study

This section empirically illustrates the above indicators by using anonymised and aggregated crime data from during the COVID-19 pandemic period.

### 4.1 M1-city (China)

The People’s Republic of China is considered the first country to experience COVID-19 [60]. It also happens to be one of the first places in the world where authorities not only introduced public-policy measures aimed at slowing or stopping contagion, but has also lifted them [61–63]. The exact beginning of the pandemic is difficult to trace. According to reports, the earliest indication of a pending epidemic available to members of the public (at the time) was a post on a Chinese social media platform (WeChat) issued from a hospital in Wuhan on 1 January 2020 indicating that there was a pneumonia-like disease of unknown origins. The same day, Chinese authorities shut down Huanan Seafood Wholesale Market, from which a number of cases were found to have emanated from (*BBC News*, 2020). The People's Daily, a Chinese newspaper, referred to COVID-19 for the first time on 21 January and measures being introduced by the government in an effort to curb its spread. Lockdown measures (expected to be the greatest source of societal disruption) followed shortly after (late January, early February) for most Chinese cities. The measure experienced a high degree of compliance owing to strict enforcement supported by multiple means of detection and deterrence [64]. China began easing restrictions in a staggered approach with, for example, the majority of schools reopening on 16 March, public transport returning on 28 March, and the restriction on internal travel being lifted on 8 April 2020. Thus routine activity has largely resumed across China with few localised exceptions [61]. Although life has not completely returned to normal, many of the criminogenic conditions that were disrupted appear to have recovered to pre-COVID-19 patterns. Consequently, various cities in China provide a suitable setting to draw data from in an effort to illustrate the proposed concepts and metrics.

For our illustrative example, victimisation data for retail theft was compiled from one of the largest cities (11 m. pop., 8000 km^2^, urbanization rate: 80%) in China. Before the pandemic, the city had one of the highest GDP in the country and an unemployment rate around 3%. For anonymity, it is referred to as ‘M1-city’ throughout. According to Chinese criminal law retail theft is a form of theft that involves entering and stealing from business premises [65]. (Not to be confused with the Western category of the same name, this crime type is referred to as ‘commercial burglary’ in China.) It was selected for analysis specifically because the number of potential theft opportunities is known to have greatly reduced during the COVID-19 outbreak period.

### 4.2 Material and method

The dataset contains the daily number of retail thefts recorded by the police in M1-city between 26 September 2017 to 29 April 2020 (N = 947 days). Twelve different forecasting models (Mean, Naive, Drift, Auto Regressive Integrated Moving Average (ARIMA), Seasonal Naive, Holt-Winters, Seasonal Auto Regressive Integrated Moving Average (SARIMA), State Space Error, Trend, Seasonal Exponential Smoothing (ETS), Exponential Smoothing State Space Model with Box-Cox transformation, ARMA errors, Trend and Seasonal components (TBATS), a Neural Network (NNETAR), as well as KNN-multiple input multiple output, and KNN-recursive) were tested to identify the approach with the greatest predictive accuracy for this particular time-series. The time-series data was partitioned into training (26 September 2017–2 August 2019, 707 days) and test (3 August 2019–31 December 2019, 120 days) datasets and the forecasting model that produced the least error over this 120-day test time horizon was identified for use. A novel ranking procedure for forecasting approaches using Data Envelope Analysis [66] identified TBATS [67] as the model which generated the least error (RMSE = 6.7, MAE = 5.4, MASE = 0.31) and was then applied to a subset of the data (the first 827 days) to generate the counterfactual (Y_F_) between 1 January 2020 and 29 April 2020 (120 days). The theoretical model presented in Fig 3 was then fitted to ND(n), the difference between Y_F_ and Y_A_. No smoothing filter was applied to the data as this would have affected the pattern of crime. Without definitive knowledge of what caused crime to drop in this period, it was not possible to specify the date of the Disruptive Event (*t*_*DE*_). However, 1 January 2020 was selected as the initial point (*t*_*i*_) because of the potential coincidence between the public’s awareness of an outbreak in a hospital in Wuhan as indicated above. The time horizon for the analysis, *t*_*h*_, was selected based on the availability of data at the time of writing and also because it yielded a four-month long study period. All other dates for the theoretical model (*t*_*DP*,_ *t*_*LP*,_ *t*_*AP*,_ *t*_*rP*_ and *t*_*RP*_) were estimated by applying a multiple change points detection method [68] and fitting the seven-phase model described above to the crime difference, ND(n). The result was then used to estimate the six indicators that were also introduced in the previous section.

### 4.3 Results

**Fig 5:**
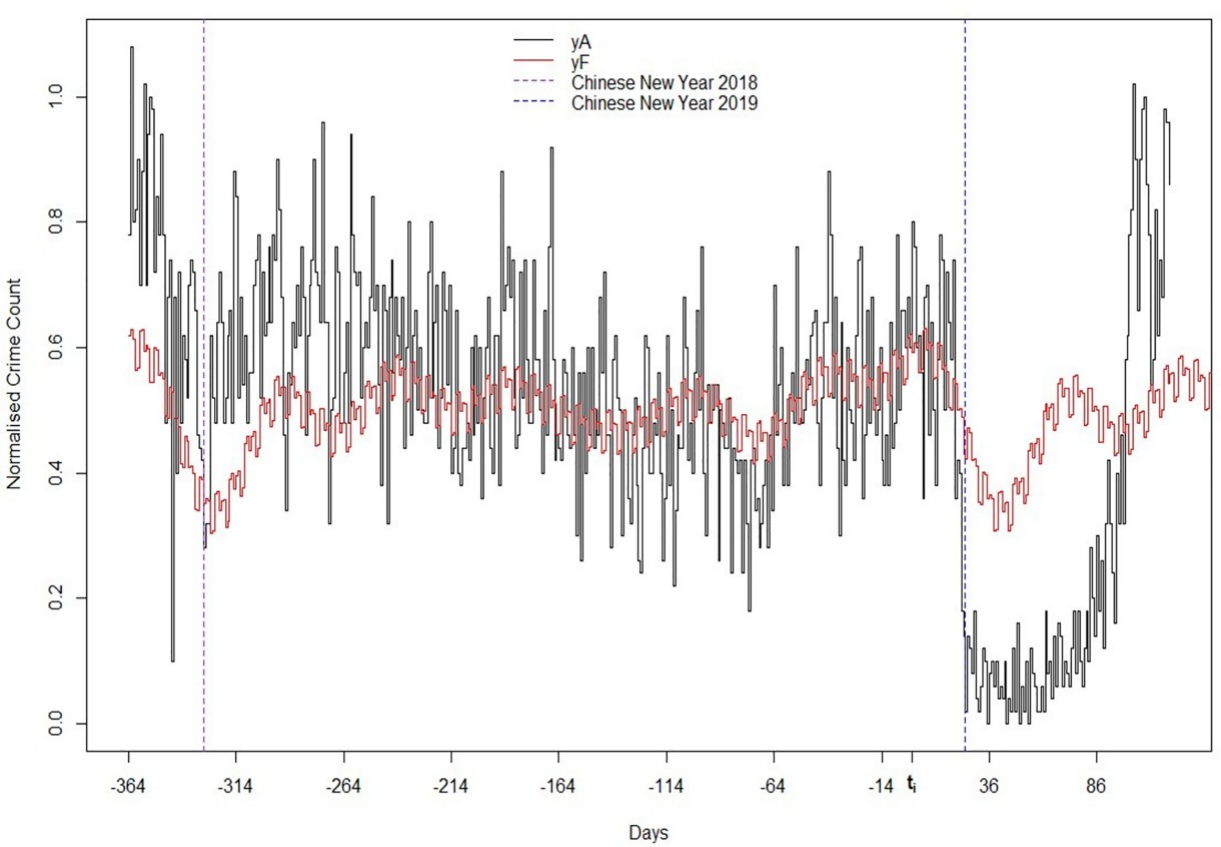
Normalised daily counts of theft in M1-city (1 January 2019–29 April 2020): Actual (Y_A_/max(Y_A_)) and predicted (Y_F_/max(Y_F_)).

Fig 5 shows the daily number of retail thefts dropped to almost zero in January 2020 before recovering several months later. The unprecedented magnitude of the reduction along with the timing strongly suggests that the COVID-19 crisis had a major effect on offenders in M1-city. Furthermore, the resemblance with the dipper-shaped curve in Fig 2 provides support for the theoretical model presented in the first section. This pattern should, however, not be taken at face value and the drop entirely attributed to COVID-19 and associated measures. Indeed, closer analysis of the time-series reveals a general trend with decreasing annual crime levels (-20 per cent between 2018 and 2019). In addition, a more localised dip was found to occur each year at the time of the Spring festival, potentially related to the low crime levels in the same month (-16 and -12 per cent compared to the following month in 2018 and 2019, respectively). This annual dip is a critical feature of the time-series as the introduction of stringency measures across cities in China coincided with the official dates for the 2020 Spring festival (25 January– 4 February). The generation of a realistic counterfactual time-series that can capture secular trends and seasonal components is therefore essential for estimating the net effect of the COVID-19 pandemic and associated measures. The forecasted time-series (Y_F_) was considered a good model for this. Not only was the accuracy within the acceptable domain but it also predicted a drop in crime at roughly the same time as the 2020 Spring festival holiday (25–30 January).

The normalised difference in crime levels (Eq 7) was then examined more closely to determine the different inflection points in the time-series. The stochastic models generated from it by the multiple change points detection method provided both a range of possible dates for each discontinuity and the shape of the resilience curve. The shape of the resilience curve generated from the stochastic method is provided in S1 Fig. A consequence of the large variance during both the first and third phases meant that finding the precise time that difference levels departed from their previous state with absolute certainty was not achievable. The algorithm provided a range of dates from as early as 16 and as late as 22 for the departure point (*t*_*DP*_). Sensitivity analysis led us to adopt the 20 January 2020 for model fitting (*τ*^*S*^ = 19 days). The same strategy was adopted for identifying *t*_*LP*,_ *t*_*AP*,_ and_,_ *t*_*RP*_ from the stochastic models. However, the overall shape of the resilience curve was less sensitive to small variations of these points. Of particular interest is the ascent point (31 March 2020) when crime levels started to increase again.

**Fig 6:**
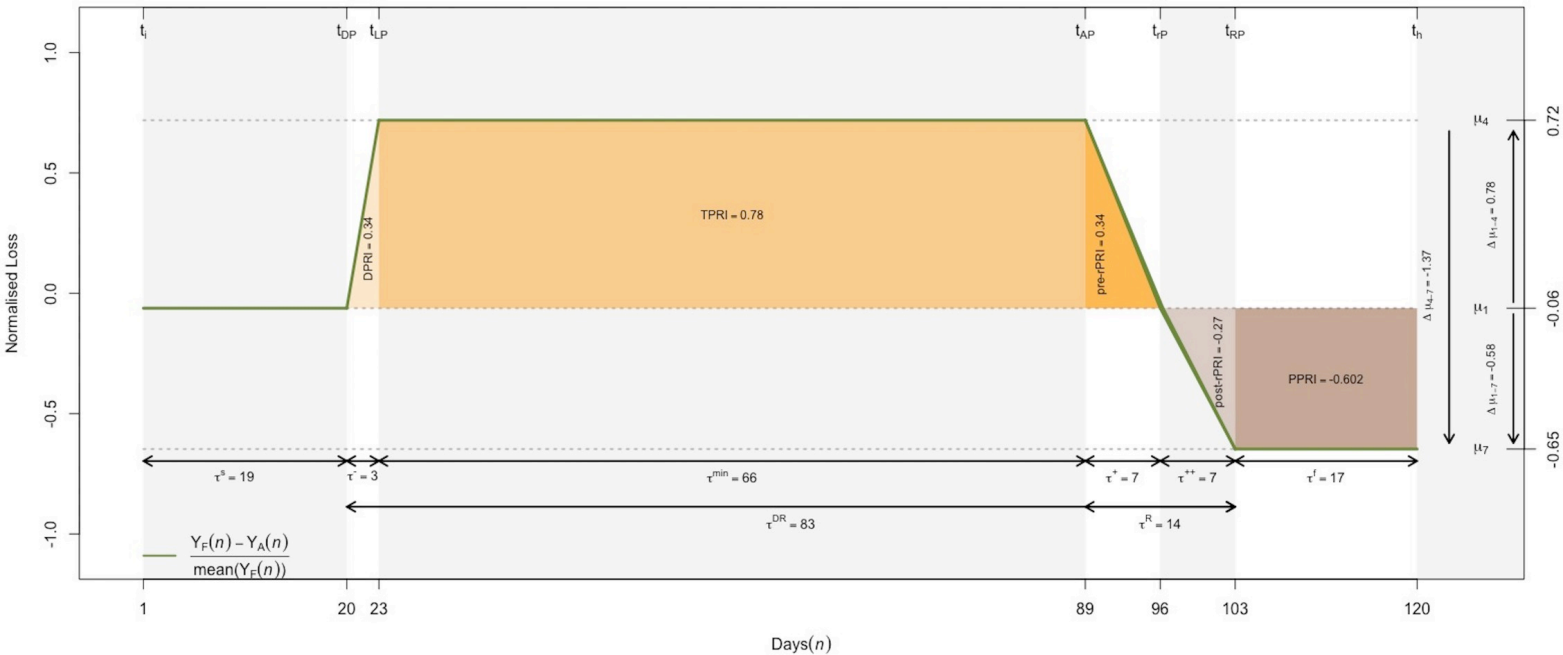
Resilience difference indicator and corresponding indicators and measures in M1-city (1 January 2020–29 April 2020).

Fig 6 is a graphical display of the resilience curve generated from the data derived from the stochastic model. The period between the departure point (*t*_*DP*_) and recovery point (*t*_*RP*_) lasted from day 20 to 103 (*τ*_*DR*_ = 83 days). Within this period, the damping phase was the shortest one (*τ*^−^ = 3 days), suggesting the criminogenic ecosystem was poorly resilient. However, this result should be interpreted with caution because, as noted above, there were multiple alternatives identified as a potential departure point. For example, the classification of *t*_*DP*_ to an earlier date (e.g., 18 January) would imply a slightly more gradual effect on crime. The trough was the longest phase of the model (*τ*^*min*^ = 66 days), with the recovery phase only starting more than two months (*τ*^−^+*τ*^*min*^ = 69 days) after the departure point. The recovery phase (*τ*^*R*^ = 14 days) was split between a pre-restoration phase (*τ*^+^ = 7 days) where crime levels returned to the pre- *t*_*DP*_ level, and a post-restoration phase (*τ*^++^ = 7 days) prior to a new equilibrium being found.

The resilience indicators (summarised in S1 Table) calculated based on the resilience curve generated from the stochastic method, hereafter referred to as the linear *ND* model, are very close to those directly estimated from the data, and easier to visually interpret. Comparison between *τ*^−^ and *τ*^+^ shows it took significantly longer for crime to drop than to return to its expected level. The large difference between DPRI (0.33) and pre-rPRI (0.4) further suggests that the cumulative loss in crime performance was higher during the restoration phase than in the damping phase. The high value of TPRI (0.78) shows the criminogenic ecosystem had limited resiliency during the trough phase. While some thefts were recorded during this period, the numbers fell to 30 per cent of the expected level (μ_4_ = 0.7).

Although the number of thefts is expected to stabilise in the near future (if they have not already), it is worth noting that the average post-recovery level is 47 per cent higher than pre-COVID-19 levels corresponding to a negative change Δμ_1–7_ = -0.60. This phenomenon may be due to several factors discussed in the section that follows. Despite this, the cumulative counts suggest there was an overall reduction in theft, with an estimated 883 incidents that did not occur (36 per cent fewer than the expected total) during this period. Assuming retail theft rates stay at the level seen in the post-recovery (posterior) phase, the cumulative loss in crime would be null by the 177^th^ day (26 June). However, this is not certain as it may return to its pre-*t*_*DP*_ level before that date.

### 4.4 Interpretation and limitations

Several criminogenic factors influencing retail theft risk were likely affected by communications regarding the COVID-19 outbreak and/or the lockdown measures imposed to tackle it. While it is not possible to draw any definitive conclusions regarding these from this data, a number of plausible explanations exist. It is conceivable, for example, that some store managers decided to fit stores with additional, or more effective security, during this period. Increased natural surveillance inside stores that remained open, primarily grocery and other essentials, might have had a suppressing effect on levels of theft as customers were afforded fewer opportunities for deception. Reduced in-store footfall and greater inter-customer distance might have facilitated detection of shoplifters. Formal surveillance by the authorities is likely to have increased too, as part of their general public order activities. This is especially likely as many Chinese municipalities have recently deployed dense CCTV networks that can be used for public safety and security [69, 70]. With few people in the streets, those who attempted to steal may have been more easily identified and arrested (or deterred) by the police. Similarly, the number of individuals commuting from outside the city able to commit theft would be diminished with restrictions on travel.

The crime drop observed after the point of departure supports the general hypothesis that the COVID-19 crisis had a significant impact on retail theft and potentially public safety more generally. The fact that COVID-19 cases in China are traced back to late 2019 suggests the main source of disruption to offenders was not the virus itself, but rather awareness of the outbreak (unofficial announcement of a respiratory disease of unknown origins occurred on 1 January while an official announcement of human-to-human transmission was made on the 20 January 2020) and/or contagion measures imposed to control the spread of the virus.

The overall shape of the resilience curve suggests it may take time for offenders to adapt their behaviour, and provides further empirical support for the notion that offenders are (relatively) rational agents who weigh the associated costs and benefits of committing an offense. Routine activity and the perception of risk, reward and effort associated with offending are therefore likely to have played an important role in the damping and recovery phases. Police agency and media reports suggest that similar drops have occurred in other countries. However, scientific inquiry is necessary to compare patterns in different places. While restrictions on travel and various other prevention measures meant to curb the spread of COVID-19 remain in place in most countries around the world, it is too early to compare the restoration phase found herein with developments elsewhere.

## 5. Discussion

### 5.1 Potential applications of the resilience indicators

While on the one hand the limited research conducted in relation to criminality in the face of disaster may be positive as it is an indicator of how infrequently as a species we have had to face potential existential crises, on the other, it has limited the efforts made by the security and safety science community of researchers to quantify and better understand the crime and disorder patterns that emerge during such disasters so that measures can be put in place to prevent particular suboptimal outcomes. This article, including the resilience indicators contained within it, provides one of the first efforts (of which the authors are aware) to describe, quantitatively, how a given criminogenic ecosystem functions throughout such a disaster. Of course, describing what has occurred creates a set of expectations grounded in empirical evidence. The indicators provide a way to capture a return to optimal functionality, as illustrated in the case of retail theft in M1-city. Further, they can inform policy-makers responsible for the allocation of resources during disaster periods, police and private security professionals, and business owners, that they may face a bounce-back in criminal activity, which they may be able to pre-empt with security measures before restrictions on public life are eased.

Retail theft in M1-city in China, is one of any number crime categories that can be examined using the indicators developed in this paper. Indeed, these non-performance metrics are applicable to any category of crime and disorder during a disaster and can be used to analyse the same category of crime within cities, and across other geographic scales. Thus, they enable comparisons to be made between different urban environments such as M1-city, but also compare urban patterns with rural ones. The metrics developed in this paper are crime-, disaster-, and resolution-agnostic enabling any category of crime, for any disaster, at any geographic scale to be described. The approach opens up the opportunity for cross-national comparisons of the impact of COVID-19 and the associated stringency measures, as well as at the state-, province-, or county-level, and even at the city- and neighbourhood-levels.

Interestingly, the indicators also offer a unique opportunity to analyse the resilience of offenders and the other constellation of actors that exist within the criminogenic ecosystem (e.g., human victims, property targets, guardians, and other types of informal and formal controllers). These metrics may give rise to more complex questions related to what has ultimately driven particular patterns to emerge throughout the disaster period. For example, if the rate of crime receded over a longer period of time in A1-City than in B1-City, was it a function of victims in A1-City not modifying their behaviour in relation to the stringencies that impacted the ecosystem; or, conversely, was it that offenders in B1-City adapted to the new ecological equilibrium more rapidly.

Similarly, and in line with what was found in M1-City, is the bounce-back that was described found elsewhere? While the indicator cannot provide a clear indication of whether the bounce-back was the result of offender adaptation or a lack of preparedness on the part of business owner to modify or adopt new public safety and security measures, the latter seems the more likely of the two. The coincidence of stringency orders being lifted that opened up internal mobility, coupled with the reopening of schools and public transport, suggests that it was a lack of preparedness on the part of commercial businesses in relation to their susceptibility to retail theft in this new normal that emerged from the disaster period. An alternative explanation may be that with the lifting of stringencies comes a period of hyper-criminality among hardened offenders who perhaps felt pressure to make up for lost opportunities to offend emanating from the disaster. Either scenario suggests that this is something that will have to be followed closely as disaster periods end across the globe and a return to normalcy (or a new normal) sets in.

It is important to recognise the limitations of the findings reported here. It is possible that the pattern observed in M1-City is unique, describing a trend that is not found anywhere else in China, or other cities around the world. We believe this is unlikely given the magnitude, spread, and nature of the COVID-19 stringency measures that have been adopted throughout the world and the associated anecdotal evidence that has repeatedly trickled into media reports about the general decline of many crimes during this period. This being said, it may be that some of the metrics are misleading.

Let us consider the recovery of retail theft in M1-city as an example. It may be that what we describe is not a function of offender adaptation, hyper-criminality after a period of criminogenic deprivation, or even a lack of preparedness by commercial businesses to retail theft, but rather a reporting error generated as a function of a return to normalcy. More specifically, much has been written about the temporal nature of crime reporting and the challenge of identifying the period in which a criminal event occurs. In fact, methods have emerged in the criminological literature in an effort to overcome this limitation (see, for example, [71]). Hence, the apparent bounce-back may be an artefact of a large number of businesses reporting incidents they learned of when stringencies were lifted. However, we believe that the large disparity in the cumulative count of expected versus actual retail theft events that occurred during the disaster period makes this unlikely. Future studies of the same crime category across different scales and geographies should be conducted to more formally test this potentiality.

Further possible limitations relate to recording practices and the confluence of stringency orders. Like the crime reporting bounce-back artefact noted above it may be that a large, disproportionate number of arrests were made during the ‘lockdown’ period when individual movement was largely prohibited. Future studies examining changing patterns of arrest to explore the extent to which these individuals have existing criminal records, may throw some light on this.

The phenomenon of offender self-selection [72], whereby those committing more serious offences also commit more minor ones, may mean that those liable to commit crimes such as retail theft receive enhanced police attention as breaches in restrictions in everyday activity are vigorously enforced during lockdowns. Again research on police activity and arrest patterns may be able to examine whether this is the case.

Finally, as noted earlier in this paper, domestic violence appears to have increased rather than decreased during lockdown, due to the enforced confinement of families in contrast to of types of offence. This will require its own analysis.

## 6. Conclusion

The resilience indicators introduced in this paper provided a set of tools to quantify the (in)ability of an ecosystem to maintain a certain level of criminal activity. Demonstrated through a case-study, it constitutes an important benchmark for an area of public safety research that requires significant attention as recently called for in the resilience literature as it relates the current pandemic (see [73]). Indeed, it fills a gap as no unifying theoretical framework existed prior to this work for describing the impact of disasters on criminogenic ecosystems and public safety (see [74]). What is gleaned from the approach can ultimately aid in the development of a greater understanding of what can be expected during disasters and consequently will enable those involved in the practice and policy of public safety to better anticipate needs.

The extant public safety literature that has focused on criminality during disasters has relied heavily upon more ubiquitous inferential methods that ordinarily hinder like-for-like comparisons. Herein lies the novelty and contribution of what has been developed herein. These indicators are reproducible, replicable, and comparable regardless of setting or scale. Consequently, the framework will enable a more refined understanding of the evolution (and resilience) of criminogenic ecosystems and public safety during the COVID-19 pandemic and may be useful for other future disasters.

## Supporting information

S1 Fig *ND* model and *ND* linear model change points extrapolated from the stochastic method. (TIF)

S1 Table Retail theft resilience indicators (M1-city, 1 January 2020–29 April 2020). *indicators used to calculate the resilience curve generated from the stochastic model.
(DOCX)
