# Supplementary figures and images for: Measuring the resilience of criminogenic ecosystems to global disruption: A case-study of COVID-19 in China
Source: PLoS One. 2020 Oct 14;15(10):e0240077. doi: 10.1371/journal.pone.0240077 (PMC7556819; doi:10.1371/journal.pone.0240077)

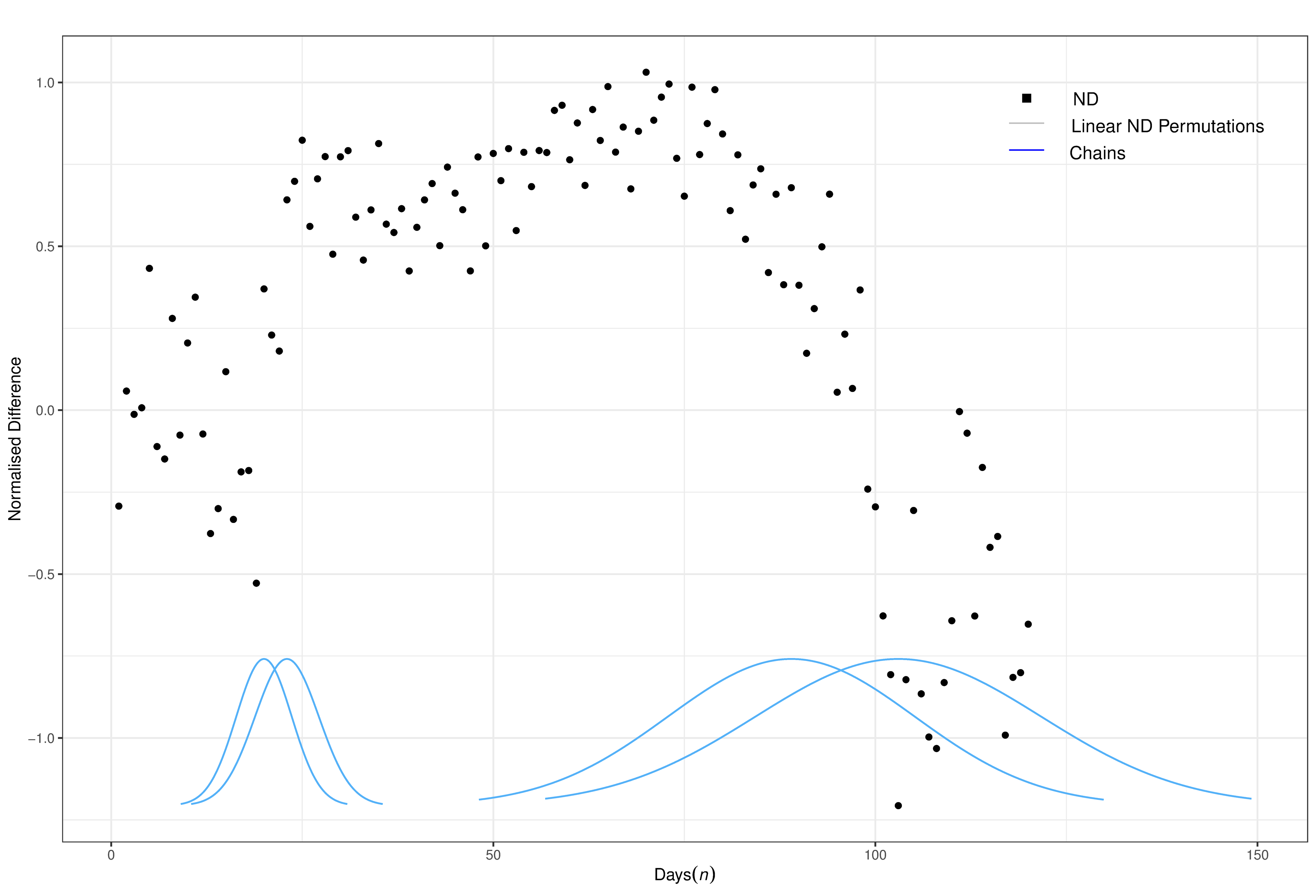

Supplement: S1 Fig — (TIF) [file pone.0240077.s001.tif]
