# Supplementary material for: Measuring the resilience of criminogenic ecosystems to global disruption: A case-study of COVID-19 in China
Source: PLoS One. 2020 Oct 14;15(10):e0240077. doi: 10.1371/journal.pone.0240077 (PMC7556819; doi:10.1371/journal.pone.0240077)
Supplement: S1 Table — *indicators used to calculate the resilience curve generated from the stochastic model. (DOCX) [file pone.0240077.s002.docx]

| **Phase onset** | | | **Phase duration** | | | **Magnitude** | | | **Resilience indicators** | | | |
| --- | --- | --- | --- | --- | --- | --- | --- | --- | --- | --- | --- | --- |
| $t_{i}$ | = | 1 | $\tau^{s}$ | = | 19 |  |  |  |  |  | **Data** | **Stochastic*** |
| $t_{DE}$ | = | ? | $\tau^{◊}$ | = | ? | $\mu_{1}$ | = | -0.11 | RDRI | = | 0.30 | 0.31 |
| $t_{DP}$ | = | 20 | $\tau^{-}$ | = | 3 | $\mu_{4}$ | = | 0.70 | DPRI | = | 0.24 | 0.33 |
| $t_{LP}$ | = | 23 | $\tau^{min}$ | = | 66 | $\mu_{7}$ | = | -0.53 | TPRI^*^ | = | 0.78 | 0.78 |
| $t_{AP}$ | = | 89 | $\tau^{+}$ | = | 7 |  |  |  | RPRI | = | 0.11 | 0.04 |
| $t_{rP}$ | = | 96 | $\tau^{++}$ | = | 7 | $\Delta\mu_{1-4}$ | = | 0.81 | pre-rPRI | = | 0.40 | 0.36 |
| $t_{RP}$ | = | 103 | $\tau^{R}$ | = | 14 | $\Delta\mu_{4-7}$ | = | -1.23 | post-rPRI | = | -0.19 | -0.28 |
| $t_{h}$ | = | 120 | $\tau^{DR}$ | = | 83 | $\Delta\mu_{1-7}$ | = | -0.41 | PPRI^*^ | = | -0.60 | -0.60 |
|  |  |  | $\tau^{f}$ | = | 17 |  |  |  |  |  |  |  |
